# Impact of constipation on patients with schizophrenia and depression: an internet-based survey (CONSIDER survey)

**DOI:** 10.3389/fpsyt.2026.1904119

**Published:** 2026-10-05

**Authors:** Hikaru Hori, Hiroyoshi Takeuchi, Shinji Yoneda, Tadashi Shin, Yusuke Shimada, Minami Umeyama, Masato Ueno, Yoshiteru Takekita

**Affiliations:** 1 Department of Psychiatry, Faculty of Medicine, Fukuoka University, Fukuoka, Japan; 2 Department of Psychiatry, Graduate School of Medicine, Chiba University, Chiba, Japan; 3 Medical Department, EA Pharma Co., Ltd., Tokyo, Japan; 4 Pharmaceutical Development Department, EA Pharma Co., Ltd., Tokyo, Japan; 5 Department of Neuropsychiatry, Faculty of Medicine, Kansai Medical University, Osaka, Japan

**Keywords:** constipation, depression, patient preference, quality of life, schizophrenia, work productivity and activity impairment

## Abstract

**Background:**

Constipation, a common gastrointestinal disorder, impairs quality of life (QOL) and work productivity. This study evaluated constipation severity in individuals with schizophrenia, depression, and the general population and examined its impact on QOL and work productivity, as well as patient education and preferences regarding constipation management.

**Methods:**

A cross-sectional web-based survey was conducted. Assessments included the Constipation Scoring System (CSS), Short Form-8, Work Productivity and Activity Impairment questionnaire, and questions regarding prior explanations of constipation risk and changes in bowel habits. CSS scores were stratified as ≤5 and ≥6.

**Results:**

In total, 800 participants completed the survey (schizophrenia, n=200; depression, n=200; general population, n=400). CSS scores were higher in the schizophrenia and depression groups than in the general population. Multivariable regression analysis showed that schizophrenia and depression were associated with constipation severity. In the schizophrenia and depression groups, current symptom status and anxiolytic use remained independently associated with CSS scores. Across all groups, participants with CSS scores ≥6 had lower Mental Component Summary scores and higher levels of presenteeism, overall work productivity loss, and activity impairment than those with CSS scores ≤5. Approximately 30% of all participants in each of the schizophrenia and depression groups reported receiving prior explanations regarding psychotropic-related constipation risk, while 40% reported bowel-habit changes after psychotropic drug initiation.

**Conclusions:**

Constipation severity was higher in participants with schizophrenia and depression than in the general population; greater severity was associated with lower QOL and greater work productivity impairment. Routine screening, education on constipation, and patient-centered constipation management may be useful clinical approaches for addressing constipation and its associated burden in these populations.

**Trial registration:**

This study was registered with the University Hospital Medical Information Network (UMIN000058981).

## 1 Introduction

Constipation is a common gastrointestinal disorder that causes abnormal bowel movements and impairs quality of life (QOL) (1). The prevalence of functional constipation is estimated at 10.1% according to the Rome IV diagnostic criteria (2), and the prevalence of self-reported constipation is generally higher than that estimated using the Rome criteria (3). Factors contributing to constipation include age, sex, socioeconomic status, dietary intake, physical inactivity, comorbidities, medications, and mental health status (4–6). In particular, schizophrenia and depression frequently coexist with constipation (7–9), partly because of reduced daily activity and the use of antipsychotics, antidepressants, and benzodiazepines (10, 11). In addition, dysregulation of the gut-brain axis has been suggested to be involved in gastrointestinal symptoms, such as constipation, in patients with psychiatric disorders, including schizophrenia and depression (12, 13). Among antipsychotics, clozapine has been strongly associated with gastrointestinal hypomotility and constipation (14, 15). Severe constipation can progress to ileus or intestinal ischemia and become life-threatening; therefore, prevention, early detection, and early treatment are crucial (16, 17).

Constipation, schizophrenia, and depression have each been associated with reduced QOL (18–20) and work productivity (21, 22). Patients with schizophrenia or depression who also have constipation may experience further impairments in QOL and productivity compared with those without constipation. Untreated constipation in patients with depression or anxiety disorders affects work productivity and QOL (23). However, the effects of constipation severity on the QOL and work productivity of patients with schizophrenia remain unclear. Furthermore, few existing reports have focused on the severity and management of constipation or medication preferences for its treatment in patients with schizophrenia and depression (17, 24).

The CONstipation in patients with SchIzophrenia and DEpRession (CONSIDER) survey was designed to clarify constipation severity in patients with schizophrenia and depression, as well as its association with QOL and work productivity. This study examined constipation severity and management, its impact on QOL and work productivity, and patient preferences regarding treatment among Japanese participants with schizophrenia, those with depression, and the general population.

## 2 Materials and methods

### 2.1 Participants

Data from a web-based survey conducted in October 2025 among Japanese residents with schizophrenia, those with depression, and the general population were utilized. The survey was administered by Cross Marketing, Inc., a Japanese online research company.

Eligible participants were panel members who self-reported having schizophrenia, depression, or neither condition (general population) and provided informed consent to participate. Information regarding whether participants had received a psychiatrist-confirmed diagnosis was not collected. No exclusion criteria were applied. The target sample size was based on a feasibility assessment. This assessment indicated that approximately 200 participants with schizophrenia could be recruited from the survey panel. Therefore, the target sample size was set at 200 participants with schizophrenia, 200 participants with depression, and 400 participants from the general population as a reference group. In each group, recruitment was closed once the target number of responses had been collected.

Ethical approval was granted by the Yamauchi Clinic Research Ethics Committee (approval no. 2025-08-00363; Tokyo, Japan), and the study adhered to the Declaration of Helsinki. This study was registered with the University Hospital Medical Information Network (UMIN000058981).

### 2.2 Survey design

The email invitations contained unique URLs. First, the research purpose was explained, and electronic informed consent was obtained before participants proceeded to the questionnaire. Next, participants reported their mental health status (schizophrenia, depression, or neither) and provided demographic information, including sex, age, employment status, and smoking status. Those who self-reported schizophrenia or depression also provided information about age of onset, type of medical institution, visit frequency, current symptom status (1, very good; 2, relatively good; 3, neither good nor bad; 4, relatively poor; and 5, very poor.), use of antipsychotics or antidepressants, use of hypnotics and anxiolytics, whether their psychiatrist explained the risk of constipation prior to initiating antipsychotic or antidepressant treatment, and changes in bowel movements after starting these medications. All participants were surveyed regarding their QOL, work productivity, and constipation severity. The survey system prevented duplicate responses, and incomplete responses could not be submitted.

### 2.3 Short Form-8 health survey (SF-8)

QOL was measured using the SF-8, which assesses eight health domains—physical functioning, role physical, bodily pain, general health, vitality, social functioning, role emotional, and mental health—and yields Physical Component Summary (PCS) and Mental Component Summary (MCS) scores (25).

### 2.4 Work Productivity and Activity Impairment Questionnaire: General Health (WPAI-GH)

Work productivity was assessed using survey data from the WPAI-GH. The WPAI-GH measures the extent to which health problems affect work and daily life over the past 7 days, including absenteeism, presenteeism, overall work productivity loss, and activity impairment (26). Assessments of absenteeism, presenteeism, and overall work productivity loss applied only to employees. Higher WPAI-GH scores indicate greater impairment.

### 2.5 Constipation Scoring System (CSS) and Bristol Stool Form Scale (BSFS)

Constipation severity was measured using the CSS, which quantifies symptom severity and is widely used in Japan (27, 28). The CSS comprises eight variables: frequency of bowel movements, painful evacuation effort, feeling of incomplete evacuation, abdominal pain, time in lavatory per attempt, type of assistance in evacuation, unsuccessful attempts at evacuation per 24 hours, and duration of constipation (27). Each variable is scored from 0 to 4, except for type of assistance in evacuation, which is scored from 0 to 2. Accordingly, the global score ranges from 0 to 30, with higher scores indicating greater severity. Stool consistency was assessed using the BSFS and categorized as hard stools (types 1–2), normal stools (types 3–5), and soft stools (types 6–7) (29).

### 2.6 Additional survey items

Participants with a CSS score ≥1 were asked about their constipation status, including constipation symptoms, urge to defecate, and abdominal bloating, as well as their satisfaction with constipation treatment. All participants were asked about their preferred method of medication administration and their expectations and concerns regarding laxatives. The details are provided in **Supplementary Table 1**.

### 2.7 Statistical analysis

Continuous variables were summarized as means and categorical variables as the number of cases (%). Demographic and clinical characteristics (sex, age, duration of illness, employment status, smoking status, medical institution, and current symptom status) were assessed using standardized mean differences (SMD). Unpaired t-tests were performed to compare continuous variables between the schizophrenia or depression groups and the general population. Within each group, unpaired t-tests were performed to compare PCS, MCS, and WPAI scores between participants with CSS scores ≤5 and those with CSS scores ≥6. Categorical variables were compared by performing the chi-square test. Trend analysis was conducted using the Jonckheere–Terpstra test (one-sided) to evaluate whether constipation severity increased monotonically with worsening current symptom status. Multivariable regression analyses were conducted to examine factors associated with CSS in all participants. CSS was modeled as the dependent variable, and sex, age, and group (schizophrenia, depression, and general population [reference]) were entered as explanatory variables. In the subgroup analyses of schizophrenia and depression, CSS, PCS, and MCS were modeled separately. For CSS, the following explanatory variables were included: sex, age, current symptom status (ordinal variable), use of drugs with anticholinergic effects, use of hypnotics, and use of anxiolytics. The following explanatory variables were used for PCS and MCS: sex, age, current symptom status (ordinal variable), CSS score, use of drugs with anticholinergic effects, use of hypnotics, and use of anxiolytics. The following drugs were defined as having anticholinergic effects: quetiapine, olanzapine, clozapine, chlorpromazine, haloperidol, perphenazine, fluphenazine, tricyclic antidepressants, and tetracyclic antidepressants. A p-value <0.05 was considered statistically significant. Because this study was exploratory, no adjustment for multiple comparisons was performed. All analyses were performed using the SAS software, version 9.4 (SAS Institute Japan Ltd.). A CSS score of ≥6 was considered indicative of moderate constipation severity (30). Because no validated CSS cutoff is available for psychiatric populations, this threshold was used to stratify participants according to constipation severity, and associations with SF-8 and WPAI-GH were evaluated. PCS and MCS were analyzed by categorizing BSFS into hard, normal, and soft stools.

## 3 Results

### 3.1 Participants

Emails were sent to panel members registered with Cross Marketing, Inc. who self-reported schizophrenia (n=956), depression (n=605), or no psychiatric symptoms (general population, n=3507). Consent was obtained from 200 participants with schizophrenia, 200 participants with depression, and 400 participants from the general population. The demographic and clinical characteristics are summarized in **Table 1**. The proportions of male participants were 66.0% in the schizophrenia group, 46.0% in the depression group, and 46.3% in the general population (SMD = 0.274). Mean ages (± SD) were 49.4 ± 9.7, 51.7 ± 10.8, and 53.7 ± 13.3 years, respectively (SMD = 0.255). In participants with schizophrenia, the most commonly used antipsychotic was risperidone (25.0%), followed by olanzapine (20.0%) and aripiprazole (17.5%). In participants with depression, serotonin-norepinephrine reuptake inhibitors were the most commonly used antidepressants (33.0%). Details of psychotropics taken by participants are shown in **Supplementary Tables 2** and **3**.

**Table 1:** Demographic and clinical characteristics.

Characteristic	Schizophrenia (n=200)	Depression (n=200)	General population (n=400)	SMD
Sex (male), n (%)	132 (66.0%)	92 (46.0%)	185 (46.3%)	0.274
Age, years	49.4 ± 9.7	51.7 ± 10.8	53.7 ± 13.3	0.255
Duration of illness, years	18.0 ± 10.3	15.2 ± 9.4	–	0.288
Employment status, n (%)				0.514
Full-time	48 (24.0%)	55 (27.5%)	185 (46.3%)	
Part-time	37 (18.5%)	30 (15.0%)	81 (20.3%)	
Student	1 (0.5%)	0 (0.0%)	5 (1.3%)	
Full-time homemaker	16 (8.0%)	25 (12.5%)	48 (12.0%)	
Others	18 (9.0%)	9 (4.5%)	10 (2.5%)	
Unemployed	80 (40.0%)	81 (40.5%)	71 (17.8%)	
Smoking status, n (%)				0.156
Never	106 (53.0%)	105 (52.5%)	219 (54.8%)	
Current	46 (23.0%)	55 (27.5%)	74 (18.5%)	
Former	48 (24.0%)	40 (20.0%)	105 (26.3%)	
Medical institution, n (%)				0.455
University hospital	14 (7.0%)	10 (5.0%)	–	
General hospital	79 (39.5%)	50 (25.0%)		
Clinic	97 (48.5%)	138 (69.0%)		
Not receiving treatment	10 (5.0%)	2 (1.0%)		
Current symptom status, n (%)				0.467
Very good	42 (21.0%)	13 (6.5%)	–	
Relatively good	67 (33.5%)	63 (31.5%)		
Neither good nor bad	44 (22.0%)	63 (31.5%)		
Relatively bad	33 (16.5%)	41 (20.5%)		
Very bad	13 (7.0%)	20 (10.0%)		
Laxative, n (%)				–
Magnesium oxide	33(16.5%)	27 (13.5%)	11 (2.8%)	
Elobixibat	4 (2.0%)	4 (2.0%)	1 (0.3%)	
Lubiprostone	6 (3.0%)	4 (2.0%)	2 (0.5%)	
Linaclotide	2 (1.0%)	0 (0.0%)	0 (0.0%)	
Polyethylene glycol	1 (0.5%)	3 (1.5%)	1 (0.3%)	
Lactulose	0 (0.0%)	1 (0.5%)	0 (0.0%)	
Stimulant laxatives	19 (9.5%)	10 (5.0%)	5 (1.3%)	
Kampo	11 (5.5%)	10 (5.0%)	2 (0.5%)	
Suppositories	1 (0.5%)	2 (1.0%)	0 (0.0%)	
Enema	1 (0.5%)	2 (1.0%)	0 (0.0%)	
Others	1 (0.5%)	1 (0.5%)	1 (0.3%)	
Over-the-counter drugs	13(6.5%)	14 (7.0%)	45 (11.3%)	
Unknown	2 (1.0%)	3 (1.5%)	2 (0.5%)	

Data are shown as numbers (%) or mean ± standard deviations.

SMD, Standardized Mean Difference.

### 3.2 CSS and BSFS

CSS scores were significantly higher in participants with schizophrenia (Cohen’s d = -0.466) and those with depression (Cohen’s d = -0.541) than in the general population (**Figure 1A**). The percentages of participants with CSS scores ≥6 were 56.0% in the schizophrenia group, 55.0% in the depression group, and 35.8% in the general population (**Figure 1B**). The self-reported current symptom status was significantly associated with CSS scores (**Supplementary Figure 1**). In the multivariable regression analysis of the full sample, sex, schizophrenia, and depression were independently associated with constipation severity (**Table 2**). In the subgroup analyses of the schizophrenia and depression groups, current symptom status and anxiolytic use remained independently associated with CSS scores (**Table 3**). The percentages of participants with a BSFS score of 4 were relatively lower in the schizophrenia and depression groups than in the general population (**Supplementary Figure 2**).

**Figure 1:**
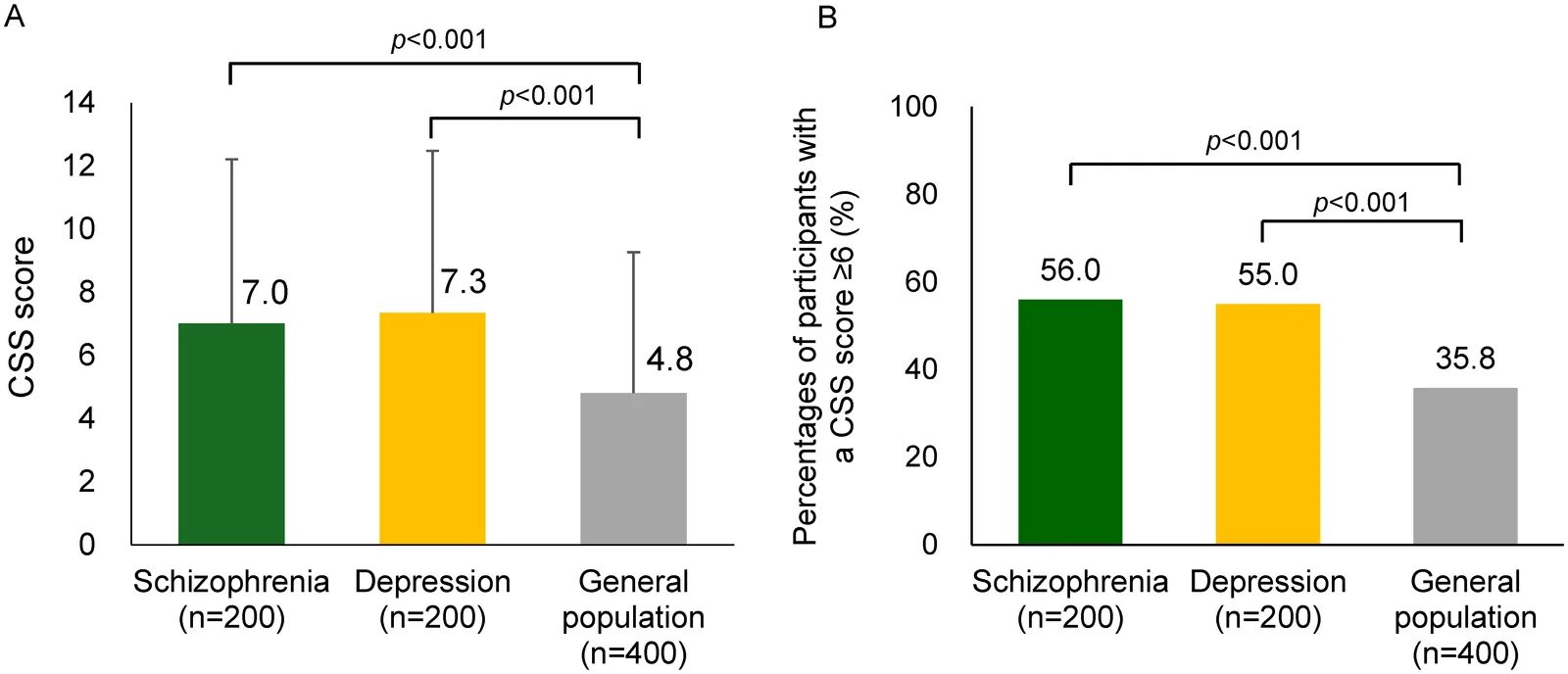
Constipation scoring system scores in schizophrenia, depression, and the general population. Constipation Scoring System Scores **(A)** and percentages of participants with Constipation Scoring System ≥6 **(B)**. Data are presented as mean ± standard deviation or percentage. CSS, Constipation Scoring System.

**Table 2:** Multivariable regression analysis for the constipation scoring system across all groups.

Variable	Coefficient	Standard error	*p*-value	95% CI	VIF	Adjusted R^2^
Sex (female)	0.939	0.353	0.008	0.246, 1.631	1.079	0.065
Age, years	-0.017	0.015	0.262	-0.045, 0.012	1.071	
Schizophrenia	2.319	0.429	<0.001	1.477, 3.161	1.130	
Depression	2.510	0.417	<0.001	1.692, 3.329	1.195	

CI, confidence intervals; VIF, variance inflation factor.

**Table 3:** Multivariable regression analysis for the constipation scoring system in participants with schizophrenia and those with depression.

Schizophrenia	Coefficient	Standard error	*p*-value	95% CI	VIF	Adjusted R^2^
Sex (female)	0.685	0.760	0.369	-0.815, 2.184	1.042	0.081
Age, years	0.025	0.038	0.513	-0.049, 0.099	1.057	
Current symptom status	1.049	0.306	0.001	0.445, 1.652	1.066	
Use of drugs with anticholinergic effects	-1.112	0.763	0.146	-2.617, 0.392	1.056	
Use of hypnotics	0.780	0.788	0.323	-0.774, 2.334	1.243	
Use of anxiolytic	1.641	0.808	0.044	0.047, 3.235	1.242	
Depression	Coefficient	Standard error	*p*-value	95% CI	VIF	Adjusted R^2^
Sex (female)	0.821	0.722	0.257	-0.603, 2.245	1.041	0.055
Age, years	-0.036	0.034	0.245	-0.106, 0.027	1.064	
Current symptom status	0.831	0.339	0.015	0.162, 1.501	1.091	
Use of drugs with anticholinergic effects	1.120	1.115	0.317	-1.080, 3.320	1.056	
Use of hypnotics	-0.227	0.782	0.772	-1.768, 1.315	1.192	
Use of anxiolytic	1.603	0.767	0.038	0.090, 3.115	1.170	

CI, confidence intervals; VIF, variance inflation factor.

### 3.3 Influence of constipation severity on SF-8

The PCS and MCS scores of participants with schizophrenia and those with depression were significantly lower than those of the general population (**Supplementary Figure 3**). Within both schizophrenia (Cohen’s d = -0.477) and general population groups (Cohen’s d = -0.644), participants with CSS scores ≥6 had significantly lower PCS scores than those with CSS scores ≤5 (**Figure 2A**). Across all groups, participants with CSS scores ≥6 also had significantly lower MCS scores than those with CSS scores ≤5, with Cohen’s d values of -0.420, -0.650, and -0.474 for the schizophrenia, depression, and general population groups, respectively (**Figure 2B**). In the schizophrenia group, participants with hard stools (BSFS types 1–2) had lower PCS and MCS scores than those with normal stool types (**Supplementary Figure 4**). In the multivariable regression analysis (**Table 4**), sex, current symptom status, and CSS scores in the schizophrenia group were independently associated with PCS, while age, current symptom status, CSS scores, and use of anxiolytics were associated with MCS. In the depression group, current symptom status and CSS scores were independently associated with PCS scores, whereas age, current symptom status, and CSS scores were independently associated with MCS scores.

**Figure 2:**
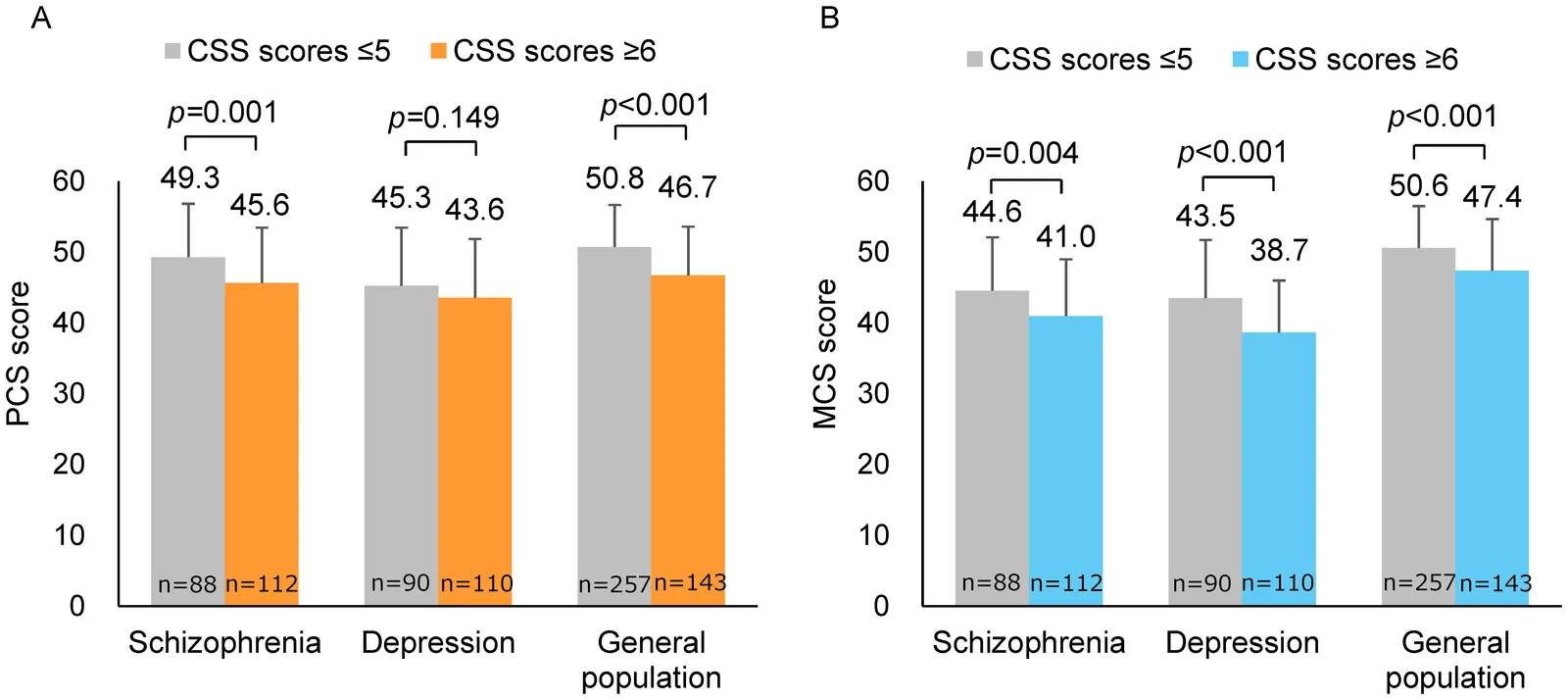
Physical component summary score and mental component summary score by constipation scoring system score. Physical component summary score **(A)** and mental component summary score **(B)**. Data are presented as mean ± standard deviation. CSS, Constipation Scoring System; MCS, Mental Component Summary; PCS, Physical Component Summary.

**Table 4:** Multivariable regression analysis for Physical Component Summary and Mental Component Summary scores in participants with schizophrenia and those with depression.

Schizophrenia	Coefficient	SE	*p*-value	95% CI	VIF	Adjusted R^2^
PCS						
Sex (female)	-2.891	1.054	0.007	-4.970, -0.812	1.046	0.233
Age, years	-0.020	0.052	0.695	-0.123, 0.082	1.060	
Current symptom status	-2.302	0.436	<0.001	-3.162, -1.442	1.123	
CSS	-0.364	0.100	<0.001	-0.560, -0.167	1.131	
Use of drugs with anticholinergic effects	0.196	1.061	0.853	-1.897, 2.289	1.068	
Use of hypnotics	-1.312	1.093	0.231	-3.468, 0.843	1.250	
Use of anxiolytic	-0.028	1.130	0.981	-2.257, 2.202	1.269	
MCS						
Sex (female)	1.420	1.014	0.163	-0.580, 3.420	1.046	0.415
Age, years	0.191	0.050	<0.001	0.092, 0.289	1.060	
Current symptom status	-3.641	0.419	<0.001	-4.468, -2.814	1.131	
CSS	-0.225	0.096	0.020	-0.414, -0.036	1.123	
Use of drugs with anticholinergic effects	0.726	1.021	0.478	-1.288, 2.740	1.068	
Use of hypnotics	-1.398	1.051	0.185	-3.471, 0.676	1.250	
Use of anxiolytic	-2.689	1.087	0.014	-4.833, -0.544	1.269	
Depression	Coefficient	SE	*p*-value	95% CI	VIF	Adjusted R^2^
PCS						
Sex (female)	-1.274	1.026	0.216	-3.297, 0.749	1.048	0.270
Age, years	-0.071	0.048	0.144	-0.166, 0.024	1.072	
Current symptom status	-3.323	0.488	<0.001	-4.285, -2.360	1.125	
CSS	-0.251	0.102	0.014	-0.452, -0.050	1.092	
Use of drugs with anticholinergic effects	-2.010	1.583	0.206	-5.132, 1.113	1.062	
Use of hypnotics	0.441	1.107	0.691	-1.742, 2.624	1.193	
Use of anxiolytic	-2.074	1.098	0.060	-4.239, 0.092	1.197	
MCS						
Sex (female)	-0.359	0.831	0.667	-1.997, 1.280	1.048	0.467
Age, years	0.096	0.039	0.015	0.019, 0.173	1.072	
Current symptom status	-4.463	0.395	<0.001	-5.243, -3.683	1.125	
CSS	-0.244	0.083	0.004	-0.406, -0.081	1.092	
Use of drugs with anticholinergic effects	1.000	1.283	0.437	-1.530, 3.530	1.062	
Use of hypnotics	-0.417	0.897	0.642	-2.186, 1.351	1.193	
Use of anxiolytic	0.414	0.890	0.642	-1.340, 2.169	1.197	

CI, confidence intervals; CSS, Constipation Scoring System scores; MCS, Mental Component Summary; PCS, Physical Component Summary; SE, Standard error; VIF, variance inflation factor.

### 3.4 Influence of constipation severity on WPAI-GH

The number of employed participants in the schizophrenia, depression, and general population groups was 95 (47.5%), 87 (43.5%), and 266 (66.5%), respectively. Compared with the general population, participants with schizophrenia and those with depression showed greater absenteeism, presenteeism, overall work productivity loss, and activity impairment (**Supplementary Figure 5**). Within each group, participants with CSS scores ≥6 exhibited significantly greater presenteeism (Cohen’s d = 0.514, 0.695, and 0.663 for the schizophrenia, depression, and general population groups, respectively), overall work productivity loss (Cohen’s d = 0.554, 0.653, and 0.655, respectively), and activity impairment (Cohen’s d = 0.591, 0.548, and 0.626, respectively) compared with those with CSS scores ≤5 (**Figures 3A–D**).

**Figure 3:**
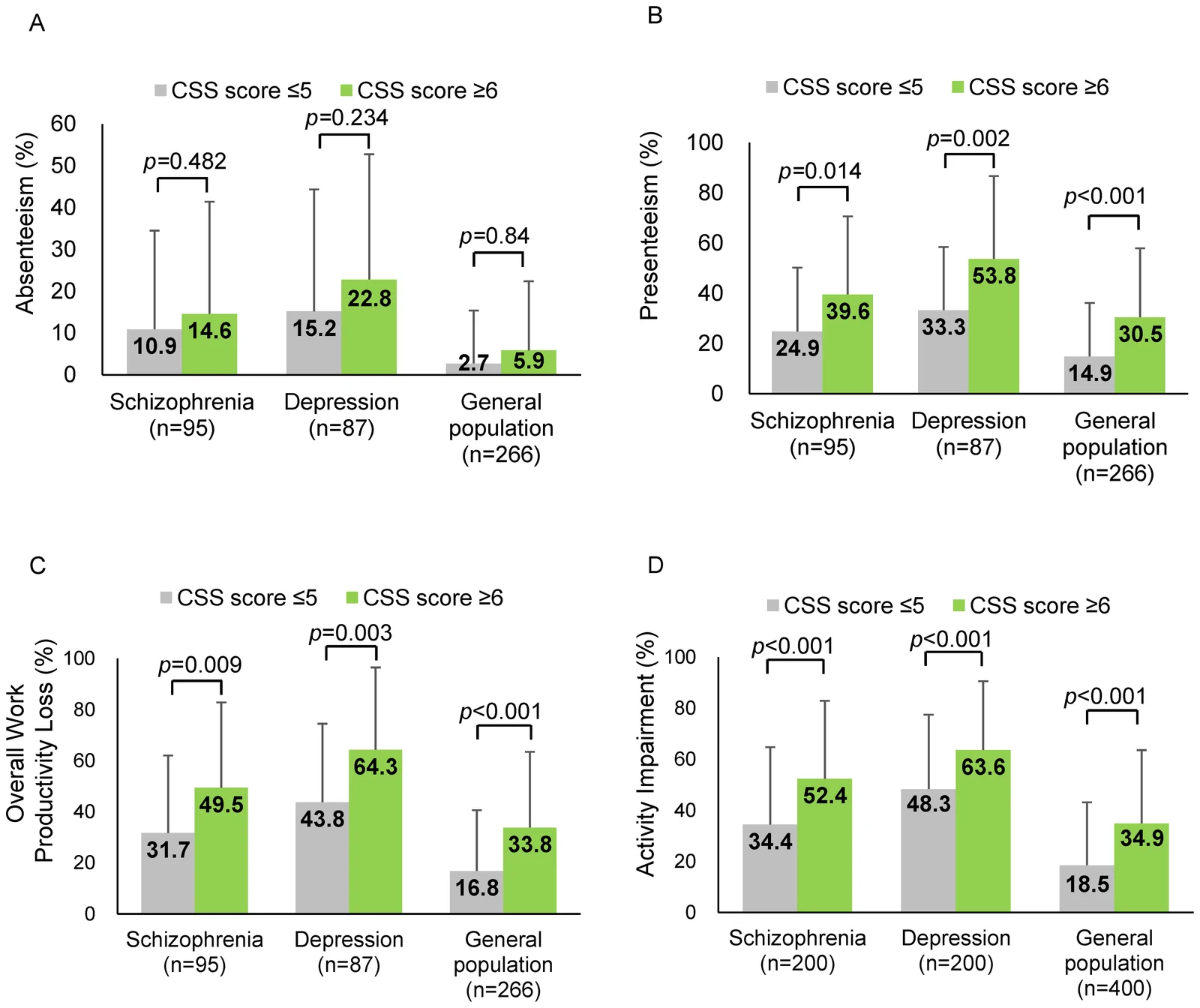
Work productivity and activity impairment by constipation scoring system score. Absenteeism **(A)**, presenteeism **(B)**, overall work productivity loss **(C)**, and activity impairment **(D)**. Data are presented as mean ± standard deviation. CSS, Constipation Scoring System.

### 3.5 Explanation of constipation risk and changes in bowel movements

Approximately 30% of participants with schizophrenia and those with depression reported being informed that constipation may occur as an adverse effect of antipsychotics or antidepressants; conversely, approximately 50% reported receiving no such explanation (**Figure 4A**). Among participants with schizophrenia, 43.5% reported changes in bowel habits after initiating antipsychotic medication, whereas 42.0% reported no changes. In the depression group, 32.0% reported changes in bowel habits after initiating antidepressants, whereas 54.5% reported no changes (**Figure 4B**).

**Figure 4:**
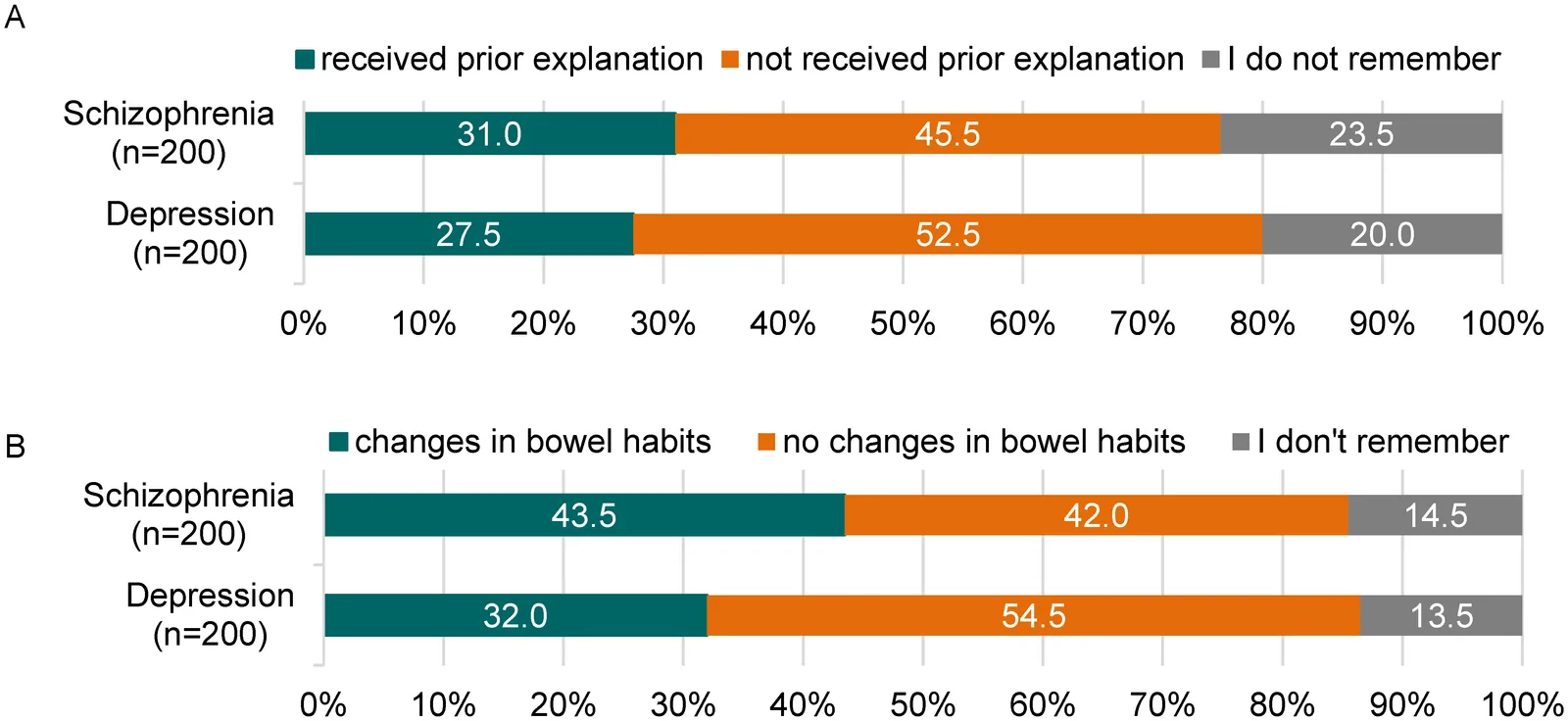
Prior explanation and bowel-habit changes related to psychotropics. Percentages of patients receiving prior explanations of constipation risk **(A)** and reporting post-initiation changes in bowel habits after starting antipsychotics or antidepressants **(B)**.

### 3.6 Management of constipation, laxative use, and medication preferences

The results regarding laxative use and preferences are presented in **Supplementary Figures 6**–**8**. Among participants with CSS scores ≥1 (schizophrenia, n=184; depression, n=190; general population, n=323), daily laxative use was reported by 24.5%, 21.1%, and 5.9%, respectively; laxative use only when no bowel movement occurred was reported by 15.8%, 10.5%, and 14.6%, respectively. Among laxative users (schizophrenia, n=74; depression, n=60; general population, n=66), prescription laxatives were more frequently obtained in the schizophrenia and depression groups, whereas over-the-counter laxatives were more commonly purchased in the general population. The most common reason for choosing over-the-counter laxatives was inconvenience of visiting medical institutions. Reasons for not using laxatives among participants with a CSS score ≥1 included the following: symptoms not severe enough to require medication, financial burden, concerns about side effects, and difficulty purchasing medication or visiting a medical institution. The preferred formulation of laxatives was tablets. The preferred dosing timing was once daily at bedtime, followed by once daily before meals.

## 4 Discussion

In this study, we found that the proportion of participants with CSS scores ≥6 was higher in the schizophrenia and depression groups than in the general population. Across all groups, participants with CSS scores ≥6 had lower MCS scores and higher levels of presenteeism, overall work productivity loss, and activity impairment than those with CSS scores ≤5. Notably, only approximately 30% of the participants reported receiving prior explanation regarding constipation risk associated with psychotropics, and approximately 40% noted changes in bowel habits after initiating these medications.

In Japanese surveys, the mean CSS scores were 4.1 for the general population and 6.8–8.6 for individuals with depression (23, 31), values similar to those observed in this study. Higher CSS scores were observed in participants with schizophrenia and those with depression than in the general population; to our knowledge, this is one of the first studies to assess the severity of constipation in participants with schizophrenia. These associations remained independent after multivariable adjustment. A high prevalence of constipation among patients with schizophrenia and those with depression has been reported (7–9, 23), which is consistent with the constipation severity observed in this study. The high prevalence of constipation in these disorders has been attributed to adverse effects of psychotropics, genetic overlap, insufficient fluid intake, low dietary fiber, and reduced physical activity (8, 10, 11, 32, 33). Among participants with schizophrenia and those with depression, worse current symptoms were associated with higher CSS scores and remained independently associated with CSS. Prior studies have shown that the likelihood of coexisting constipation increases as depression and anxiety severity worsens (23), whereas depressive symptoms alone are associated with constipation (34, 35). Because this survey was cross-sectional, causality cannot be inferred; however, the symptom status of patients with schizophrenia or depression may influence the severity of constipation. In contrast to previous studies, no independent association between the use of drugs with anticholinergic effects and CSS scores was observed. Similar null findings have been reported (36). Further research is needed to clarify the relationship between anticholinergic exposure and constipation severity. Anxiolytic use remained independently associated with CSS scores. The relationship between anxiety and constipation remains uncertain. In psychiatric outpatients, no significant association between antianxiety drugs and laxative use or defecation was observed (37). In contrast, coexisting anxiety has been reported to be associated with constipation in patients with schizophrenia (38). In this study, the observed association may have reflected anxiety related sympathetic activation and dysregulation of the hypothalamic-pituitary-adrenal axis, rather than a direct pharmacologic effect of anxiolytics (8, 38, 39). However, the mechanisms underlying this association have not been sufficiently investigated and this explanation remains speculative. The observed association may also have reflected indication bias, whereby patients with greater psychiatric symptom severity were more likely to receive anxiolytics.

Participants with schizophrenia had lower QOL than the general population, and CSS scores ≥6 were associated with further reductions in PCS and MCS scores. Additionally, abnormal stool consistency was associated with lower QOL compared with normal stool consistency, consistent with previously reported findings in patients with chronic constipation (1). In the depression group, CSS scores ≥6 were associated with a further decline in MCS scores, whereas no significant change was observed in PCS scores. Chronic constipation reportedly impairs both physical and mental health, with relatively pronounced mental impairment (40–42). Depressive symptom severity has been reported to mediate the association between constipation severity and mental health-related QOL, but not physical health-related QOL (24). In this study, constipation severity was associated with lower MCS scores in the depression group. However, because of the cross-sectional design, the direction and underlying mechanisms of this association remain unclear. Age was associated with higher MCS scores, consistent with previous findings in psychiatric populations (43). However, the relationship between age and mental health-related QOL in psychiatric populations remains unclear and warrants further investigation. Evidence regarding the effects of constipation on work productivity in patients with schizophrenia is limited, whereas untreated constipation has been linked to reduced productivity in patients with depression and anxiety (23). Based on the QOL and WPAI results, greater constipation severity was associated with poorer QOL and greater work productivity impairment among participants with schizophrenia and those with depression, suggesting that constipation severity may represent an important clinical concern in these populations.

Only approximately 30% of patients reported receiving an explanation of constipation induced by antipsychotics or antidepressants, while 40% reported changes in bowel movements after starting these medications. In a previous study, approximately half of the patients with schizophrenia were unaware of their constipation, and only 18.5% spontaneously reported it to a psychiatrist (44). Patients with mental illness may not recognize constipation, and by the time it is reported, it may be severe. Therefore, psychiatrists should provide proactive education regarding medication-induced constipation and routinely assess bowel habits as part of comprehensive care. Furthermore, participants preferred laxative tablets and once-daily dosing. This preference is similar to the reported preferences for antipsychotic formulation and dosing timing among patients with schizophrenia and psychiatrists (45). Current clinical guidelines recommend osmotic laxatives, such as magnesia and polyethylene glycol, as first-line treatment for chronic constipation (46, 47). If the current therapy is ineffective, switching to or adding a different laxative class may be considered (46, 47). In such cases, treatment plans should align with the patient’s preferences for dosing timing, convenience, and formulation (48).

This study has several limitations. First, because the data were collected through a web-based survey, selection bias toward participants with Internet access is probable, with an underrepresentation of older adults, institutionalized individuals, patients with severe psychiatric symptoms, individuals with cognitive impairment, and patients with substantial comorbidities. Therefore, the findings may not be fully generalizable to the broader population of patients with schizophrenia or depression. In addition, because this study was conducted in a Japanese population, the findings may not be directly generalizable to populations outside Japan. The employment rate of participants with schizophrenia in this study was approximately half, indicating a high proportion (49, 50), but this was comparable to the employment rate reported in previous web-based surveys of patients with schizophrenia (51). Because WPAI-GH analyses were limited to employed participants, differences in employment rates among groups may have introduced selection bias and influenced the observed work productivity outcomes. Second, schizophrenia and depression were self-reported, and diagnosis or symptom status was not confirmed by psychiatrists. In addition, psychiatric symptom severity was assessed using a simple self-rated measure rather than validated rating scales. Therefore, diagnostic misclassification and measurement bias cannot be excluded. Third, constipation was not diagnosed using the Rome IV criteria. Because individuals with schizophrenia and those with depression are likely to use laxatives more frequently, it may have been difficult to adequately assess constipation severity using the Rome IV criteria alone. Fourth, detailed information about the dose and duration of psychotropic medications and laxatives was not obtained, and some participants with high CSS scores may have experienced chronic constipation prior to initiating psychotropic treatment. In addition, psychotropic medications were categorized according to the presence or absence of anticholinergic effects, and information regarding polypharmacy and cumulative anticholinergic burden was not collected. Therefore, their potential influence on constipation severity could not be evaluated. Furthermore, the assessment of changes in bowel habits after initiating psychotropic medication relied on participants’ retrospective self-reports and may therefore have been subject to recall bias. Fifth, information on medical conditions and lifestyle factors that may influence constipation, such as gastrointestinal or endocrine disorders, body mass index, physical activity, dietary habits, and water intake, was not collected. Furthermore, smoking status, employment status, and laxative use were not included in the multivariable analyses. Therefore, these factors could not be adequately accounted for, and residual confounding cannot be excluded. Sixth, age- and sex-stratified analyses were not performed. Therefore, the potential associations of age and sex with constipation severity, QOL, and work productivity could not be fully evaluated. Seventh, because multiple statistical comparisons were performed without formal adjustment, the possibility of type I error should be considered when interpreting the findings.

## 5 Conclusions

In this study, constipation severity appeared higher among participants with schizophrenia and depression than among the general population, and greater severity was associated with lower QOL and higher work productivity impairment. Routine screening, education on psychotropic-related constipation, and patient-centred constipation management may be useful clinical approaches for addressing constipation and its associated burden in these populations.

## Data Availability

The datasets generated and/or analyzed during the current study are not publicly available due to the privacy of participants but are available from the corresponding author on reasonable request.
