# Supplementary material for: Impact of constipation on patients with schizophrenia and depression: an internet-based survey (CONSIDER survey)
Source: Front Psychiatry. 2026 Oct 5;17:1904119. doi: 10.3389/fpsyt.2026.1904119 (PMC13679139; doi:10.3389/fpsyt.2026.1904119)
Supplement: Supplementary file 1 [file SupplementaryFile1.docx]

Supplementary Material

Supplementary Figure 1. Association between current symptom status and Constipation Scoring System Scores in the schizophrenia (a) and depression (b) groups

a
b

**Supplementary Figure 2.** **Percentage of participants by Bristol Stool Form Scale**

Chi-square test, **p <0.01, ***p <0.001.

**Supplementary Figure 3.** **Physical Component Summary (a) and Mental Component Summary Scores (b) for schizophrenia, depression, and the general population**

a	b

***p <0.001.

**Supplementary Figure 4. Physical Component Summary (a) and Mental Component Summary Scores (b) by categorizing the Bristol Stool Form Scale**

**a	b**

**p* <0.05, ***p* <0.01, ****p* <0.001.

**Supplementary Figure 5. Work productivity and activity impairment in the schizophrenia, depression, and general population groups**

a	b

c	d

Absenteeism (a), presenteeism (b), overall work productivity loss (c), and activity impairment (d).

***p <0.001.

**Supplementary Figure 6. Laxative use and preferences in the schizophrenia group**

a

b	c

d	e

(Continued)

f	g

Laxative usage status (a), facility where laxatives are obtained (b), reasons for not obtaining a prescription at a medical institution (among over-the-counter purchasers) (c), reasons for not taking laxatives (among non-laxative users) (d), preferred laxative dosage form (e), preference ratings for dosing timing and frequency (f), and preferred dosing method (g) in the schizophrenia group.

**Supplementary Figure 7. Laxative use and preferences in the depression group**

a

b	c

d	e

(Continued)

f	g

Laxative usage status (a), facility where laxatives are obtained (b), reasons for not obtaining a prescription at a medical institution (among over-the-counter purchasers) (c), reasons for not taking laxatives (among non-laxative users) (d), preferred laxative dosage form (e), preference ratings for dosing timing and frequency (f), and preferred dosing method (g) in the depression group.

**Supplementary Figure 8. Laxative use and preferences in the general population**

a

b	c

d	e

(Continued)

f	g

Laxative usage status (a), facility where laxatives were obtained (b), reasons for not obtaining a prescription at a medical institution (among over-the-counter purchasers) (c), reasons for not taking laxatives (among non-laxative users) (d), preferred laxative dosage form (e), preference ratings for dosing timing and frequency (f), and most preferred dosing method (g) in the general population.

**Supplementary Table 1. Questionnaires on types and management of constipation, laxative usage, and medication preferences**

1 Please answer the following questions about your constipation and its management.
  1.1 Constipation symptoms.
      Infrequent bowel movements (no stool passes) / difficulty with defecation (inability to pass stools)
  1.2 Do you feel the urge to go to the bathroom before bowel movements?
      Usually / Sometimes / Rarely
  1.3 Do you experience abdominal bloating?
      Often / Sometimes / Rarely
  1.4 Are you making dietary changes to improve constipation?
      Yes / No
  1.5 Are you actively exercising to improve constipation?
      Yes / No
  1.6 Do you take laxatives?
      I take them almost every day / only when I cannot have a bowel movement / I do not take laxatives.
  1.7 (Shown only to respondents who do not take laxatives) Please tell us why you do not take laxatives.
      My symptoms are not severe enough to need medication / I do not want to take medication due to concerns about side effects / It is a difficult to purchase medication or visit a medical institution / It would be a financial burden / Other (free text).
  1.8 (Shown only to respondents who take laxatives: “almost every day” or “only when I cannot have a bowel movement”) How do you obtain the laxative you take?
      I purchase it at a drugstore / I visit a medical institution and receive a prescription.
  1.9 Have you experienced nausea (feeling bad) after taking laxatives?
      No / Yes
  1.10 Have you ever experienced stomach pain after taking laxatives?
       No / Yes
  1.11 Have you ever experienced diarrhea after taking laxatives?
       No / Yes
  1.12 (Shown only to respondents who purchase laxatives over the counter) Why do you not visit a medical institution for constipation?
       Visiting a medical institution is inconvenient / I feel embarrassed to see a doctor for constipation/concern about extra money / Other (free text)
  1.13 (Shown only to respondents who receive prescriptions for laxatives at a medical institution) Please select all constipation treatments you are currently taking (select at least one):
       Magnesium Oxide / Elobixibat / Lubiprostone / Linaclotide / Polyethylene glycol / Lactulose / Stimulant laxatives / Kampo / Suppositories / Enemas / Other (free text) / Unknown
  1.14 How satisfied are you with your current treatment for constipation?
       Very satisfied / Somewhat satisfied / Neither satisfied nor dissatisfied / Somewhat dissatisfied / Very dissatisfied
2 If you use laxatives, what dosage do you prefer?
   Tablets / capsules / powder to be dissolved in water / liquid / suppositories / enema.
3 Please rate your preference for each dosing frequency and timing on a 10-point scale (1 = very undesirable to 10 = very desirable).
  3.1 Once daily before meals.
  3.2 Once daily at bedtime.
  3.3 Twice daily after breakfast and dinner.
  3.4 Three times daily before or after meals.
4 Among the aforementioned dosing methods, which is the most preferable?
  3.1 / 3.2 / 3.3 / 3.4
5 The importance of the following features of constipation medicine:
  Very important/Somewhat important/Not important.
  5.1 Reliable effectiveness
  5.2 Complete evacuation (no feeling of incomplete evacuation)
  5.3 Predictable timing of bowel movements
  5.4 Easy to take
  5.5 Low incidence of side effects (nausea, diarrhea, abdominal pain)
6 Among these items, which are most important to you?
  5.1 / 5.2 / 5.3 / 5.4 / 5.5
7 Please indicate your concerns regarding the following when using laxatives.
  Not very concerned / Somewhat concerned / Quite concerned
  7.1 Feeling nauseous
  7.2 Developing diarrhea
  7.3 Abdominal pain
  7.4 Effect on the fetus during pregnancy
8 Among the concerns mentioned above, which worries you the most?
  7.1 / 7.2 / 7.3 / 7.4

**Supplementary Table 2. Psychotropics taken by participants with schizophrenia**

| Psychotropics | Schizophrenia  (n=200) | |
| --- | --- | --- |
| Aripiprazole  Quetiapine  Risperidone  Brexpiprazole  Olanzapine  Lurasidone  Clozapine  Blonanserin  Chlorpromazine  Paliperidone  Perospirone, Asenapine  Haloperidol, Perphenazine, Fluphenazine  Hypnotics  Anxiolytic  Others  Unknown  Not taking any psychotropics | 35  26  50  17  40  8  4  17  5  8  12  6  95  77  24  10  10 | (17.5%)  (13.0%)  (25.0%)  (8.5%)  (20.0%)  (4.0%)  (2.0%)  (8.5%)  (2.5%)  (4.0%)  (6.0%)  (3.0%)  (47.5%)  (38.5%)  (12.0%)  (5.0%)  (5.0%) |

**Supplementary Table 3. Psychotropics taken by participants with depression**

| Psychotropics | Depression  (n=200) | |
| --- | --- | --- |
| NaSSA  SNRI  SSRI  Tetracyclic antidepressant  Tricyclic antidepressants  Hypnotics  Anxiolytics  Others  Unknown  Not taking any psychotropics | 29  23  66  9  16  117  110  25  10  8 | (14.5%)  (11.5%)  (33.0%)  (4.5%)  (8.0%)  (58.5%)  (55.0%)  (12.5%)  (5.0%)  (4.0%) |

Data are presented as numbers (%).

NaSSA, noradrenergic and specific serotonergic antidepressants; SNRI, serotonin-norepinephrine reuptake inhibitor; SSRI, selective serotonin reuptake inhibitor
